# Comparison of Early Outcomes with Three Approaches for Combined Coronary Revascularization and Carotid Endarterectomy

**DOI:** 10.5935/1678-9741.20160076

**Published:** 2016

**Authors:** Arzu Antal Dönmez, Taylan Adademir, Hakan Sacli, Cengiz Koksal, Mete Alp

**Affiliations:** 1 Kartal Kosuyolu Yuksek Ihtisas Training and Research Hospital Istanbul, Turkey.; 2 Sakarya University Training and Research Hospital Istanbul, Turkey.

**Keywords:** Coronary Artery Bypass, Endarterectomy, Carotid, Carotid Artery Diseases

## Abstract

**Objective:**

This study aims to compare three different surgical approaches for combined coronary and carotid artery stenosis as a single stage procedure and to assess effect of operative strategy on mortality and neurological complications.

**Methods:**

This retrospective study involves 136 patients who had synchronous coronary artery revascularization and carotid endarterectomy in our institution, between January 2002 and December 2012. Patients were divided into 3 groups according to the surgical technique used. Group I included 70 patients who had carotid endarterectomy, followed by coronary revascularization with on-pump technique, group II included 29 patients who had carotid endarterectomy, followed by coronary revascularization with off-pump technique, group III included 37 patients who had coronary revascularization with on-pump technique followed by carotid endarterectomy under aortic cross-clamp and systemic hypothermia (22-27ºC). Postoperative outcomes were evaluated.

**Results:**

Overall early mortality and stroke rate was 5.1% for both. There were 3 (4.3%) deaths in group I, 2 (6.9%) deaths in group II and 2 (5.4%) deaths in group III. Stroke was observed in 5 (7.1%) patients in group I and 2 (6.9%) in group II. Stroke was not observed in group III. No statistically significant difference was observed for mortality and stroke rates among the groups.

**Conclusion:**

We identified no significant difference in mortality or neurologic complications among three approaches for synchronous surgery for coronary and carotid disease. Therefore it is impossible to conclude that a single principle might be adapted into standard practice. Patient specific risk factors and clinical conditions might be important in determining the surgical tecnnique.

**Table:** 

Abbreviations, acronyms & symbols	
**ACC**	**=Aortic cross clamp**
**CABG**	**=Coronary artery bypass grafting**
**CEA**	**=Carotid endarterectomy**
**CPB**	**=Cardiopulmonary bypass**
**ICU**	**=Intensive care unit**
**MI**	**=Myocardial infarction**
**SPSS**	**=Statistical Package for Social Science**
**TIA**	**=Transient ischemic attack**
**USAP**	**=Unstable angina pectoris**

## INTRODUCTION

Co-existence of coronary and carotid artery disease is common in clinical practice. It has been reported that the incidence of carotid artery disease in patients scheduled for coronary revascularization is 2.8%-22% and the incidence of coronary artery disease in patients scheduled for carotid endarterectomy (CEA) is 28-40%^[1,2]^. Simultaneous CEA and coronary artery bypass grafting (CABG) was first described by Bernhard et al.^[3]^, in 1972. Since then, the most efficacious and safest surgical procedure, minimizing mortality and neurological complications, has been sought, but there is still no clear consensus on the ideal approach.

The aim of this study is to compare safety and efficacy of different surgical techniques for combined CABG and CEA, with the main outcome measures being the in-hospital neurologic complications and mortality.

## METHODS

This retrospective study involves 136 patients who had concomitant CABG and CEA in our institution, between January 2002 and December 2012. The institutional review board of the hospital approved the study. Preoperative, intraoperative and postoperative data of patients were reviewed from patient records.

Mean age of patients was 65.9 years. There were 29 (21.3%) females and 107 (78.7%) males. Most of the patients (94.1%) had multivessel coronary involvement and only 8 (5.9%) patients had single vessel disease. Previous myocardial infarction (MI) was observed in 54 (39.7%) patients and 106 (77.9%) patients had unstable angina pectoris (USAP). Carotid duplex scanning was routinely performed in patients scheduled for coronary revascularization. Symptomatic patients with stenosis of more than 70% were considered for combined operation. Asymptomatic patients were further evaluated with conventional or tomographic angiography before decision making for combined surgery. Thirty two (23.5%) patients had history of stroke and 19 (13.9%) had history of transient ischemic attack (TIA), and 85 (62.5%) patients were asymptomatic. Bilateral involvement of carotid arteries was reported in 94 (69.1%) patients. Patients with permanent neurological deficit and patients having concomitant procedures besides revascularization were excluded. Demographic characteristics are presented in Table 1.

**Table 1:** Preoperative clinical characteristics.

		Group I (n=70)	Group II (n=29)	Group III (n=37)	*P*
		n (%)	n (%)	n (%)	
Age	<65	30 (42.9)	8 (27.6)	19 (51.4)	0.148
	≥65	40 (57.1)	21 (72.4)	18 (48.6)	
Gender	Male	62 (88.6)	22 (75.9)	23 (62.2)	0.006**
	Female	8 (11.4)	7 (24.1)	14 (37.8)	
Preoperative USAP		55 (78.6)	24 (82.8)	27 (73.0)	0.625
Previous MI		24 (34.3)	14 (48.3)	16 (43.2)	0.379
Multivessel coronary disease		70 (100)	23 (79.3)	35 (94.6)	0.001**
Preoperative neurological symptoms	Asymptomatic	45 (64.3)	19 (65.5)	21 (56.8)	0.661
	TIA	11 (15.7)	4 (13.8)	4 (10.8)	
	Stroke	14 (20)	6 (20.7)	12 (32.4)	
Bilateral carotid disease		50 (71.4)	21 (72.4)	23 (62.2)	0.560

Ki-kare test

** *P*<0.01

MI=myocardial infarction; TIA=transient ischemic attack; USAP=unstable angina pectoris

Preoperative neurological status of patients was defined as follows:

- Asymptomatic: without any warning neurological symptoms within 3-6 months^[4]^;
- Transient ischemic attack (TIA): a syndrome of acute neurological dysfunction referable to the distribution of a single brain artery and characterized by symptoms lasting less than 24 hours^[5]^;
- Transient stroke: neurological symptom duration between 24 hours to 7 days^[5]^;
- Stroke: focal or global neurological dysfunction that persist more than 7 days^[5]^;
- Amaurosis fugax: a sudden transient (persists for < 60 minutes) monocular visual loss^[5]^;
- Perioperative neurological event: any new sensory or motor neurological deficit, TIA, stroke^[5]^;
- Perioperative MI: elevation of myocardial enzymes (serum creatine kinase-myocardial band or troponin T level) more than 5 times the normal reference range in the first 72 hours following CABG, in association with appearence of new Q waves or left bundle brach block^[6]^.

### Surgical Technique

Patients were divided into 3 groups according to the surgical technique used:

- Group I included 70 patients underwent CEA first, followed by coronary revascularization with on-pump technique.
- Group II included 29 patients underwent CEA, followed by coronary revascularization with off-pump technique.
- Group III included 37 patients underwent coronary revascularization with on-pump technique followed by CEA under aortic cross-clamp and systemic hypothermia (22-27ºC).

In the first group of patients, operation started with CEA and saphenous vein harvesting under general anesthesia. Carotid arteries were explored in standard manner, then clamped and opened, followed by endarterectomy using a carotid shunt in most of the cases. Arteriotomy was closed directly or by using saphenous patch, then it was deaired and clamps were released. Leaving the incision open to avoid hematoma formation, cardiac part of the operation was iniated with a standard median sternotomy. After this, left internal mammarian artery was harvested, cardiopulmonary bypass was established with standard aortic arterial-unicaval venous cannulation and systemic heparinization. Body temperature was lowered to 30-32ºC. Antegrade or combined antegrade-retrograde blood cardioplegia was used for cardiac arrest and myocardial protection. After distal anastomosis was completed, cross-clamp was removed and proximal anastomosis was constructed with a partial occlusion clamp while the patient was being rewarmed. With discontinuation of cardiopulmonary bypass, heparin was reversed and both incisions were closed in routine fashion.

In the second group of patients, CEA was applied as described, followed by coronary revascularization with off-pump technique using mechanical stabilizers. Proximal anastomoses were performed with a partial occlusion clamp in an area of ascending aorta deemed free of atherosclerotic disease.

In third group of patients, surgery started with the cardiac procedure. Cardiopulmonary bypass was established in routine manner, and after this, distal anastamosis were constructed. Continuous retrograde blood cardioplegia was used and patients were cooled to a mean systemic temperature of 25ºC (22ºC-27ºC) for cerebral protection. Afterwards cardiac procedure finalization, CEA was performed in the same manner, except without using a shunt while the aorta remained clamped. The remaining part of cardiac procedure was completed while the patient was being warmed to normal systemic temperature of 37ºC.

### Statistical Analysis

Statistical analysis was performed using the Statistical Package for Social Science (SPSS) version 15.0 for Windows. Data were analysed using descriptive statistical methods (mean, standard deviation, frequency) and qualitative data were compared using Chi-square test for categorical variables. Quantitative data were analysed using Student t test for comparing intergroup differences of continous parameters. Kruskal Wallis and Mann Whitney U tests were used for comparing quantitative parameters not normally distributed between groups.

Statistical significance was assessed with *P* value less than 0.05.

## RESULTS

Preoperative characteristics were not significantly different between groups in terms of cardiac and neurological status. History of previous myocardial infarction was present in 24 (34.3%) patients in group I, 14 (48.3%) in group II and 16 (43.2%) in group III (*P*>0.05). Twenty five (35.7%) patients in group I, 10 (34.5%) in group II and 16 (43.2%) in group III had prior neurological event (TIA/stroke) (*P*>0.05). (Table 1)

The average number of grafts was 2.95 with mean aortic cross clamp (ACC) time of 59.95±24.72 minutes and cardiopulmonary bypass (CPB) time of 90.70±28.99 minutes in group I and 2.56 grafts with ACC of 91.10±40.44 and CPB time of 131.45±33.98 minutes in group III. ACC and CPB times were significantly longer in group III compared to group I (*P*<0.01). Carotid shunt was used in 95.7% of patients in group I and 93.1% of patients in group II. Carotid shunt was not used in any case in group III. There was no significant difference between groups in terms of carotid artery closure technique; in group I carotid patchplasty was prefered for 40 (57.1%) patients, in group II for 17 (58.6%) patients and in group III for 22 (59.5%) patients (Table 2).

**Table 2:** Intraoperative variables.

	Group I (n=70)	Group II (n=29)	Group III (n=37)	*P*
^+^ACC time (minute)	59.95±24.72		91.10±40.44	0.001**
^+^CPB time (minute)	90.70±28.99		131.45±33.98	0.001**
^++^Shunt used	67 (95.7)	27 (93.1)	__	0.001**
^++^Patch closure	40 (57.1)	17 (58.6)	22 (59.5)	0.972
^++^Primary closure	30 (42.9)	12 (41.4)	15 (405)	

+ Student t-test (between group I and group III)

** *P*<0.01

++ Ki-kare test

ACC=Aortic cross-clamp; CPB=Cardiopulmonary bypass

Overall early mortality was 5.1%. Cardiac events were responsible for 57.1% of mortality and 28.5% were due to neurological events. There were 3 (4.3%) deaths in group I; one was due to perioperative MI, the second was due to postoperative stroke and third one was following acute renal insufficiency complicated by infection and multiorgan failure. The 2 (6.9%) deaths in group II, one was due to MI and the other one was due to stroke. There were 2 (5.4%) deaths in group III, both were attributed to cardiac events, patients required inotrophic and intraortic baloon pump support and were lost due to low cardiac outcome syndrome. Statistically no significant difference was detected among groups in terms of mortality rates (*P*=0.860). Although ACC and CPB times were significantly longer in group III compared to group I (*P*=0.001), this difference did not contribute to mortality rates. No statistically significant difference was observed for mortality rates among the groups.

Eleven (15.7%) patients in group I, 2 (6.9%) in group II and one patient (2.7%) in group III had perioperative neurologic events, including amaurosis fugax, recurrent laryngeal nerve paralysis, extremity weakness, TIA and stroke. Although no statistical difference was analysed between three groups (*P*=0.086, *P*>0.05) for neurological events, *post-hoc* analysis between groups I and III, identified a significant difference in favor of group III (*P*=0.048, *P*<0.05).

Stroke was observed in 5 (7.1%) patients in group I. One patient, who did not awake from anesthesia, was detected to have bilateral infarcts in cranial tomography and died on the 4^th^ postoperative day. Other 4 patients were referred for early physical rehabilitation and recovered almost completely. Two (6.9%) patients in group II had stroke. One patient that had bilateral carotid lesions (one site >70% stenosis, the other site with 50-69% stenosis) and preoperative MI, had stroke on the contralateral site and this patient died on 11^th^ postoperative day. Other patient was left with minor sequelae in upper extremity. There was not any stroke observed in group III, and only one patient had recurrent laryngeal nerve palsy which recovered completely. No statistically significant difference was observed for stroke rates among the groups.

Patients in group I had longer intensive care unit (ICU) and hospital stay compared to patients in group II (*P*<0.05) in *post-hoc* analysis, but there were no significant difference between groups I and III and between groups II and III (Table 3).

**Table 3:** Postoperative outcome.

	Group I (n=70)	Group II (n=29)	Group III (n=37)	*P*
	n (%)	n (%)	n (%)	
^+^Perioperative MI	3 (4.3)	3 (10.3)	5 (13.5)	0.220
^+^IABP	__	1 (3.4)	3 (8.1)	0.061
^+^Neurological event	11 (15.7)	2 (6.9)	1 (2.7)	0.086
^+^Stroke	5 (7.1)	2 (6.9)	__	0.251
^+^Infection	10 (14.3)	__	2 (5.4)	0.050^*^
^+^Renal dysfunction	5 (7.1)	2 (6.9)	3 (8.1)	0.978
^+^Hospital mortality	3 (4.3)	2 (6.9)	2 (5.4)	0.860
^++^ICU stay (day)	4.02±3.54 (3)	2.96±2.54 (2)	3.94±3.90 (3)	0.032*
^++^Hospital stay (day)	10.01±4.64 (8)	7.86±2.72 (7)	9.45±4.61 (8)	0.061

+ Ki-kare test

++ Kruskal Wallis Test

* *P*<0.05

## DISCUSSION

Stroke is one of the major causes of morbidity and mortality after coronary revascularization^[7]^. Carotid artery disease has been shown to be an important etiological factor in postoperative stroke^[8]^. Naylor et al.^[9]^, in their review article, reported the incidence of stroke following a total of 190449 CABG to be 1.5-2.0%, but in case of co-existing severe carotid artery disease, the risk increases to 6.7%. Once happens, mortality of post-CABG stroke was reported to be 23.1%. Nwakanma et al.^[10]^ stated that adding CEA to CABG did not increase short and long term morbidity and mortality, and combined procedure can be done safely in this high risk group of patients.

Optimal surgical treatment for patients having concomitant severe coronary artery and carotid artery stenosis remains uncertain. There are different surgical techniques that may be chosen depending on the patient characteristics, severity and morphology of lesions in vascular beds, the experience and preference of the center and the surgeon. Surgical options include staged approaches in which CEA or CABG are performed following one another with a certain time interval in between and synchronous approach in which CEA and CABG are performed under same anesthesiology session. Although several studies report higher mortality and stroke rates with combined surgery compared to staged surgery^[11-13]^, most of the papers report comparable and better outcomes with synchronous procedures^[14-17]^. Outcomes of different studies documenting the results of synchronous surgery vary widely, with a stroke rate between 1% to 4% and mortality rate between 1% to 5.4%^[16,18-20]^. This variation might be due to patient profiles included in the studies (symptomatic *versus* asymptomatic). In our study stroke rate was 5.1% which is higher than would be expected, but 37.4% of our patients were symptomatic and 69.1% had bilateral involvement of carotid arteries.

Postoperative stroke is believed to be multifactorial; besides carotid artery disease, atherosclerosis of ascending aorta is an important source of emboli in on-pump CABG. Inadequate cerebral perfusion pressure, systemic vasodilatory response, risk of atheroembolization due to cannulation and aortic clamping, increase the risk of operative stroke during CPB^[21-24]^. In order to avoid these factors, some centers preferred off-pump coronary revascularization with CEA. Meharwal et al.^[22]^ reported results of their 82 patients who had combined CEA and off-pump CABG without stroke and mortality, stating off-pump CABG avoids cardiopulmonary bypass as one of the contributing factors for stroke. Two years later the same group reported that this procedure is safe and effective, with shorter intubation time, intensive care unit stay and hospital stay even in a group of patients with high risk for perioperative stroke due to aortic arch atherosclerosis, advanced age, severe ventricular dysfunction besides carotid artery disease^[24]^. The study by Gopaldas et al.^[16]^, also supported off-pump CABG to be associated with lower stroke risk than on-pump CABG in synchronous surgery group. In our clinic, off-pump CABG has been in practice since 1993, and is mainly preferred for single or double vessel revascularization. A previous study by Eren et al.^[25]^ from our center reported no stroke and 3.7% mortality for patients who had CEA and simultaneous off-pump CABG. In the literature, reported rate of mortality is between 0-4.5% and rate of stroke is between 0-3.1% for combined CEA and off-pump CABG^[16,21-24]^. In this group of patients, our mortality and stroke rates were 6.9% for both. One of the mortalities was secondary to stroke in a high risk patient with bilateral carotid lesions and recent MI who was probably operated by off-pump technique due to this profile. We did not observe any further benefit with off-pump technique compared to on-pump technique, except for shorter intensive care unit and hospitalization times.

Systemic hypothermia reduces the metabolic tissue rate and is generally employed for neuroprotection during cardiac and aortic surgeries. Khaitan et al.^[26]^ reported simultaneous CEA and CABG during single cross-clamp, under 25°C of hypothermia for further cerebral protection, as a safe technique with a mortality rate of 5.8% and stroke incidence of 5.8%. Guibaud et al.^[27]^ also stated hypothermia below 28°C provides better cerebral protection especially for patients with bilateral carotid lesions. Eren et al.^[28]^ reported the results of the first 15 patients of our center being operated under 25°C of hypothermia without stroke and mortality.

Although statistically it was not found to be an important difference among groups, there was no postoperative stroke in group III. There was a statistically significant relation between postoperative stroke and body temperature; systemic temperatures > 28°C, increase stroke risk 6.7 times (OR: 6.712; 95% CI: 0.831-54.192). In contrast to this benefit, hypothermia was found to increase risk of myocardial infarction 3.5 times (OR: 3.490; 95% CI: 0.785-15.515). This might be due to increased ACC and CPB times which were increased significantly compared to group I and it might have contributed to cardiac event related to mortality seen in this group of patients.

The main limitation of this study is its retrospective nature rather than being a prospective randomized clinical study. Patients were not divided into different surgical treatment groups depending on pre-determined criteria and the decision of using off-pump technique or moderate hypothermia was surgeon dependent. Although preoperative characteristics were not significantly different among groups in terms of cardiac and neurological status, this might have created bias.

## CONCLUSION

In this study, we aimed to compare different management techniques of synchronous surgery for coronary and carotid disease, in order to obtain a consensus regarding the best approach. Low rate of neurological events with hypothermia was de-emphasized by its detrimental cardiac effects. We found no advantage of one technique over the other; therefore it was impossible to conclude that a single principle might be adapted into standard practice. Patient specific risk factors and morphology of the atherosclerotic disease might be more important determinants of postoperative morbidity and mortality rather than the surgical technique.

**Table:** 

Authors’ roles & responsibilities	
AAD	Analysis and/or data interpretation; conception and design study; manuscript redaction or critical review of its content; realization of operations and/or trials; statistical analysis; final manuscript approval
TA	Analysis and/or data interpretation; conception and design study; manuscript redaction or critical review of its content; realization of operations and/or trials; statistical analysis; final manuscript approval
HS	Analysis and/or data interpretation; conception and design study; manuscript redaction or critical review of its content; realization of operations and/or trials; statistical analysis; final manuscript approval
CK	Analysis and/or data interpretation; conception and design study; manuscript redaction or critical review of its content; realization of operations and/or trials; statistical analysis; final manuscript approval
MA	Analysis and/or data interpretation; conception and design study; manuscript redaction or critical review of its content; realization of operations and/or trials; statistical analysis; final manuscript approval
